# Early developmental support for preterm infants based on exploratory behaviors: A parallel randomized controlled study

**DOI:** 10.1002/brb3.3266

**Published:** 2023-10-05

**Authors:** Turgay Altunalan, Zübeyir Sarı, Tuba Derya Doğan, Nilüfer Eldeş Hacıfazlıoğlu, İpek Akman, Tuğba Altıntaş, Sevil Uzer, Nihan Hande Akçakaya

**Affiliations:** ^1^ Department of Physiotherapy and Rehabilitation, Faculty of Health Science Karadeniz Technical University Trabzon Turkey; ^2^ Department of Physiotherapy and Rehabilitation, Faculty of Health Science Marmara University Istanbul Turkey; ^3^ Family Consultation Center Spastic Children's Foundation of Turkey ‐ Cerebral Palsy Turkey Istanbul Turkey; ^4^ Clinic of Pediatric Neurology, Zeynep Kamil Gynecologic and Pediatric Training and Research Hospital University of Health Sciences Istanbul Turkey; ^5^ Department of Pediatrics, Faculty of Medicine Demiroglu Bilim University Istanbul Turkey; ^6^ Department of Health Sciences Uskudar University Istanbul Turkey; ^7^ Clinic for Child Neurology and Social Pediatrics Child Centre Maulbronn Maulbronn Germany; ^8^ Department of Neurology, Faculty of Medicine Demiroglu Bilim University İstanbul Turkey; ^9^ Department of Physiotherapy and Rehabilitation, Faculty of Health Science Istinye University Istanbul Turkey

**Keywords:** early intervention, explorative behaviors, neurodevelopment, parenting, preterm infants

## Abstract

**Introduction:**

Preterm infants are at high risk for developmental disabilities, and their parents are at increased risk for high stress. Early intervention programs are applied to reduce these adverse outcomes. The primary aim is to compare the efficacy of the novel Explorer Baby early intervention program for the holistic development of preterm infants. The second objective was to compare the stress levels of their mothers.

**Methods:**

Randomized clinical trial with 38 weeks–6 months corrected age preterm infants at low risk for cerebral palsy, randomly assigned to experimental (Explorer Baby) or active control neurodevelopmental therapy (NDT) groups. Fifty‐seven infants were enrolled in the study, and 51 (26 Explorer Baby, 25 NDT) completed it. Bayley III was used as a primary outcome before, during, and after the intervention.

**Results:**

When we compared the changes between the groups before and after therapy, no significant differences were found in any of the primary or secondary outcomes (between‐group comparisons). When comparing the changes in both groups before and after therapy (in‐group comparison), the Explorer Baby group demonstrated significant improvements in cognitive (Hedges’ *g* = .83) and explorative language skills (Hedges’ *g* = .65), whereas the NDT group showed improved parent–child dysfunctional interaction (Hedges’ *g* = 2.66) between *T*0–*T*1 and *T*0–*T*2.

**Conclusions:**

The Explorer Baby early intervention program may be a preferred option to support premature infants without brain injury, as it shows greater skill acquisition than NDT, although not statistically significant. Both methods are safe as they support premature babies without negatively affecting mothers’ overall stress levels.

## Graphical abstract

The developmental trajectories of Explorer Baby and NDT follow a nearly parallel curve in language and motor skills. However, in cognitive skills, the developmental curve is significantly vertical in the Explorer Baby group.

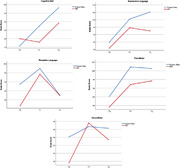

## 1 INTRODUCTION

Preterm birth is a global problem with a worldwide incidence of 10.6%, and every year, nearly 15 million preterm babies are born alive (Chawanpaiboon et al., 2019). It adversely affects infants’ neurodevelopment and their parents’ mental health (Pascal et al., 2018; Schappin et al., 2013). These adverse conditions increase gradually with the decrease in gestational age and can affect multiple areas, such as cognitive, language, fine, and gross motor development (Pascal et al., 2018). Preterm birth is an unexpected event that increases parents’ stress levels (Sabnis et al., 2019). Excessive stress interrupts parenting functions such as handling obstacles, creating free‐time activities, and reacting to infants (Winter et al., 2018). This may also adversely affect neurodevelopment (Hoge & Shaw, 2021). Early intervention programs are applied to reduce these adverse conditions and enhance infants’ global development and their parents’ well‐being (Puthussery et al., 2018). The literature suggests that early intervention programs should start as early as possible and consider parents’ active participation (Spittle & Treyvaud, 2016).

Early childhood programs for at‐risk infants have become common after understanding the sensitivity of early brain development and the importance of active experience. Although considerable efforts have been made to improve the quality of early childhood interventions, there are some limitations. The first limitation is the need for high‐quality evidence; the second is the effectiveness of traditional methods; and the third is the need for holistic interventions, including parental involvement (Benzies et al., 2013; Spittle et al., 2015). Holistic interventions are programs that support multiple areas of infant development and parental stress (Peyton et al., 2021). Holistic intervention is essential for premature infants because they are at risk for developmental delays in multiple areas, including motor, language, cognitive, and behavioral skills (Pascal et al., 2018), and their parents are at risk for increased stress and depression (Schappin et al., 2013). Additionally, current developmental theories provide new perspectives on understanding infants’ neurodevelopment. These theories highlight active experiences, environmental factors, and parents’ empowerment in early human development (Espe‐Sherwindt, 2008; Khurana et al., 2020; Kolb et al., 2017). Due to these limitations and new perspectives on early childhood development, in this study, we planned to investigate the effectiveness of a novel early intervention program called Explorer Baby Early Intervention (Explorer Baby).

### 1.1 Theoretical background of explorer baby early intervention program

Explorer Baby is a holistic developmental support program with strategies for infants at‐risk and their parents. The program is based on family‐centered practice (Dunst & Espe‐Sherwindt, 2016) and environmental enrichment in the context of dynamic system theories (Smith & Thelen, 2003). It accepts the trial and learning process in the context of neuronal group selection theories (Hadders‐Algra, 2010). Explorer Baby aims to improve caregivers’ skills in setting up play environments to increase infants’ trial‐and‐error behaviors. The unique aspect of the program is to support infants through their exploratory behaviors and motivation for movement.

The first factor is exploratory behaviors, all movements to discover themselves, toys, and surroundings. Exploratory behaviors include variable and repetitive motor and sensory activities such as gazing at an object or a person, manipulating objects, reaching, and kicking (Lobo et al., 2015). First movements start accidentally and become voluntary upon gaining active experiences (Jouen & Molina, 2005; Pocovi et al., 2019). The other factor is movement motivation, which can be described as how infants behave in the play environment. Movement motivation includes play motivation, adaptation, self‐regulation, activity skills, and exploratory behaviors. Infants with high movement motivation have a high level of participation in environmental play opportunities (Zentner & Bates, 2008). Hence, the Explorer Baby program aims to improve infants’ active experiences by increasing their exploratory and movement motivation behaviors.

### 1.2 Theoretical background of neurodevelopmental therapy (NDT/Bobath)

Dr. Karel and Mrs. Berta Bobath developed neurodevelopmental therapy (NDT) in the 1940s. The concept is updated annually. Therefore, the year in which practitioners receive NDT training is critical. In the past, it was a more passive approach where professionals would decide and teach children and parents what they needed. Currently, it involves the active participation of both children and parents. The concept relies on theories of motor control, neuromuscular plasticity, biomechanics, and motor learning to analyze human movement function, explains recovery from brain injuries, and understands posture. This approach is a client‐centered, problem‐solving, and clinical reasoning treatment for a variety of clinical conditions in pediatric groups (Mayston et al., 2023). The concept utilizes postural support and positioning techniques to provide sensory and motor stimulation to infants. It typically involves utilizing tactile, vestibular, and somatosensory cues to improve movement. NDT is characterized by therapist‐facilitated techniques to improve postural control and movement. The concept aims to improve function and skill through typical movements. Typical movements are defined as goal‐directed movements that do not lead to abnormal muscle tone or posture. The NDT program focuses on improving adaptation to the environment and developing better functional skills in infants and children by using handling, positioning, and facilitation technics, and prohibiting atypical movements or postures (Mayston, 2016).

In the current study, our first aim was to investigate the efficiency of the novel Explorer Baby early intervention program on the motor, cognitive, and language skills of preterm infants. The second aim was to examine the effect of the “Explorer Baby” early intervention program on their mothers’ stress levels.

## 2 MATERIALS AND METHODS

### 2.1 Study design and ethical considerations

This study was planned in the Department of Physiotherapy and Rehabilitation at Marmara University in Türkiye and implemented in the Family Counseling Center of Cerebral Palsy Türkiye. The design of this study was a stratified parallel‐randomized controlled study. The National Clinical Trial number is NCT04203589. tThe experimental group received the Explorer Baby early intervention program, and the active control group received NDT. Both groups received standard care in the country where the study was conducted. The assessors were blinded to the group assignment, but because of the nature of the study, practitioners and parents could not be blinded. The study's ethical approval was obtained from the Ethics Committee of Medicine Faculty of Marmara University (09.2017.729). Primary caregivers were informed, and their verbal and written consents were obtained before the initial assessment.

### 2.2 Participants

The study was conducted between June 2018 and August 2019 in Istanbul, Türkiye, and the infants were referred from a territory neonatal intensive care unit in the same city. We invited 98 parents of premature infants to the study by telephone. Of these parents, 30 refused to participate in the study, citing transportation problems to the study center and problems with taking time off from work. Researchers of this paper previously published the participation attitudes of the parents (Altunalan et al., 2020). Sixty‐eight preterm infants born before 33 weeks of gestational age and younger than the corrected age of 6 months were assessed for eligibility, and 57 infants were enrolled. All infants were referred to the study by a pediatric neurologist and a neonatologist. All of the invited parents were eligible for the study in terms of parental exclusion criteria.

**FIGURE 1:**
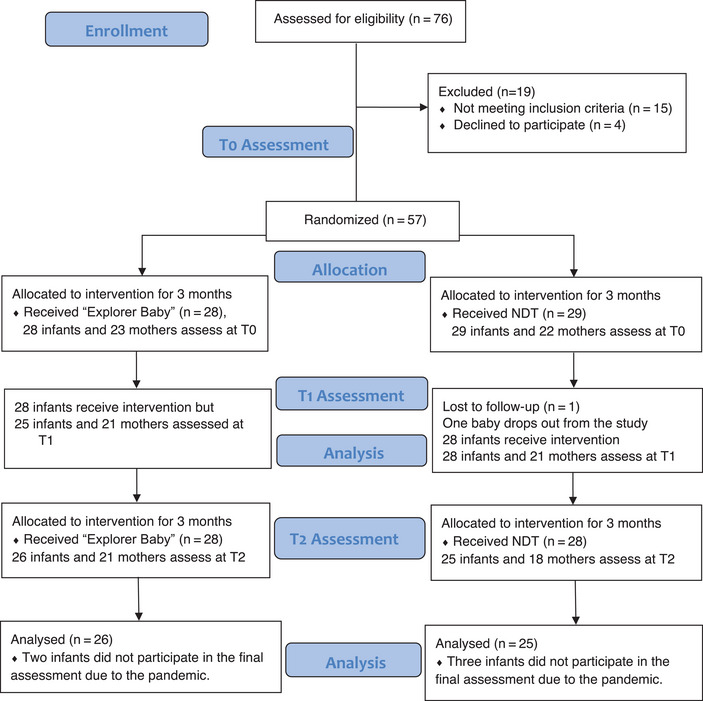
CONSORT flow diagram displaying participants’ recruitment and analysis.

We determined the eligibility of infants for the study based on their level of neurodevelopmental risk. We took infants’ biological risk factors from the Neonatal Intensive Care discharge report, whereas socioeconomic factors were recorded during parents’ interviews. The infants at a high risk of cerebral palsy (based on neurological examination, grades II, III, and IV cranial imaging, and cramped synchronized or absent fidgety movements in General Movements Analyses) (Novak et al., 2017) and with metabolic or genetic diseases were excluded from the study. If an infant had a certain diagnosis during the study, those infants were referred to another project (Figure 1). Parents who could communicate in Turkish and did not have any psychiatric diagnoses were included in the study.

Although preterm infants have a high risk of cerebral palsy and need more early intervention, we excluded those infants from our study because this was the first study investigating the effectiveness of the Explorer Baby program. After assessing the results of this initial study, the authors would like to conduct another study on infants at high risk of brain damage.

### 2.3 Randomization

As it is well known that lower gestational age and mother's education level affect neurodevelopment adversely, we stratified participants based on gestational age at birth (32—28 weeks, below 28 weeks) and the mother's educational level (primary, elementary, and bachelor's degree). Randomization was provided by the closed envelope method. Three voluntary physical therapists took part in the study. They made a random allocation sequence, enrolled participants, assigned participants to interventions, and collected data.

### 2.4 Intervention

The experimental groups received the Explorer Baby, and the active control group received NDT at the Family Counseling Center. Although the existing literature indicates that the effect of NDT on motor development is debated, we chose this method because of its widespread use in clinical practice (Elbasan et al., 2017). Both groups received standard care, which included vaccinations, screenings of hearing and vision, and routine pediatrician controls. There were no regular early intervention services in Türkiye during the time frame of the current study. Two physical therapists (17 and 12 years’ experience) who developed the program implemented Explorer Baby, and two European Bobath Tutors Association (EBTA)‐certified physical therapists implemented NDT (20‐ and 5‐years’ experience). To provide fidelity to the interventions, the therapists in the experimental group recorded their sessions with a camera and watched them together. Therapists in the NDT group worked together at baseline and periodically in sessions to ensure consistency across therapists. Because the physical therapists in the NDT group were up‐to‐date with their EBTA certification, we predicted that practitioner fidelity would be higher.

Both interventions were administered once per month for a duration of 6 months with an average session length of 45–50 min. In past studies involving premature infants without brain damage, therapy frequency ranged from once a week to once a month (Dusing et al., 2018; Spittle et al., 2009). As parents of premature babies tend to prefer attending therapies once per month (Mobbs et al., 2022), and this frequency is better suited to decreasing waiting lists in early intervention services, we designed our study to have a therapy frequency of once per month. If a therapist wanted to organize therapy more often than once a month, our working method allowed this, so that the therapy methods could be applied in a more flexible and realistic way.

#### 2.4.1 The Explorer Baby early intervention program

Explorer Baby is a family‐centered and active learning program for infants and parents. The program consists of three main contents and their sub‐contents. The first is family‐centered practices; the second is the play behaviors of infants; and the third is creating learning opportunities (environmental enrichment). We followed the family‐centered practice while communicating with the parents, especially while determining intervention goals, and understanding the parents’ priorities (Dunst & Espe‐Sherwindt, 2016). In addition, the practitioner tries to understand the cultural characteristics of parents in raising children and encourages them to find solutions in their culture.

The characteristic play content includes the infants’ adaptation, temperament, motivation, and explorative behaviors. In this context, experts and parents observe the explorative behaviors of the infant together. For example, experts and parents attempted to understand how the infant is motivated during a game, how the infant tends to stop playing or increase play/trial behaviors, what an infant does when games are too complicated, and how the infant calms down after stressful situations. In this program, parents are active and have bidirectional communication with experts.

Environmental enrichment content includes improving trial‐and‐error behaviors in motor, cognitive, language, and social fields. Experts and parents observe how the environment (toys, pillows, play positions, and strange people) affects an infant's play behaviors, and what happens when the infant sees an object.

Additionally, the Explorer Baby program has body structure issues. The expert asks themselves, “Are there any body structure issues, such as hypotonia, hypertonia, sensory, or regulation problems, that can affect an infant's play behaviors?” As the infant's exploratory behaviors may cure these findings, the standard program is applied for the first sessions. However, if these findings persist and disrupt play behaviors, the expert sets up additional therapy sessions to address these issues (Figure 2) (File S1).

FIGURE 2Practical guideline for explorer baby.

**Figure:**
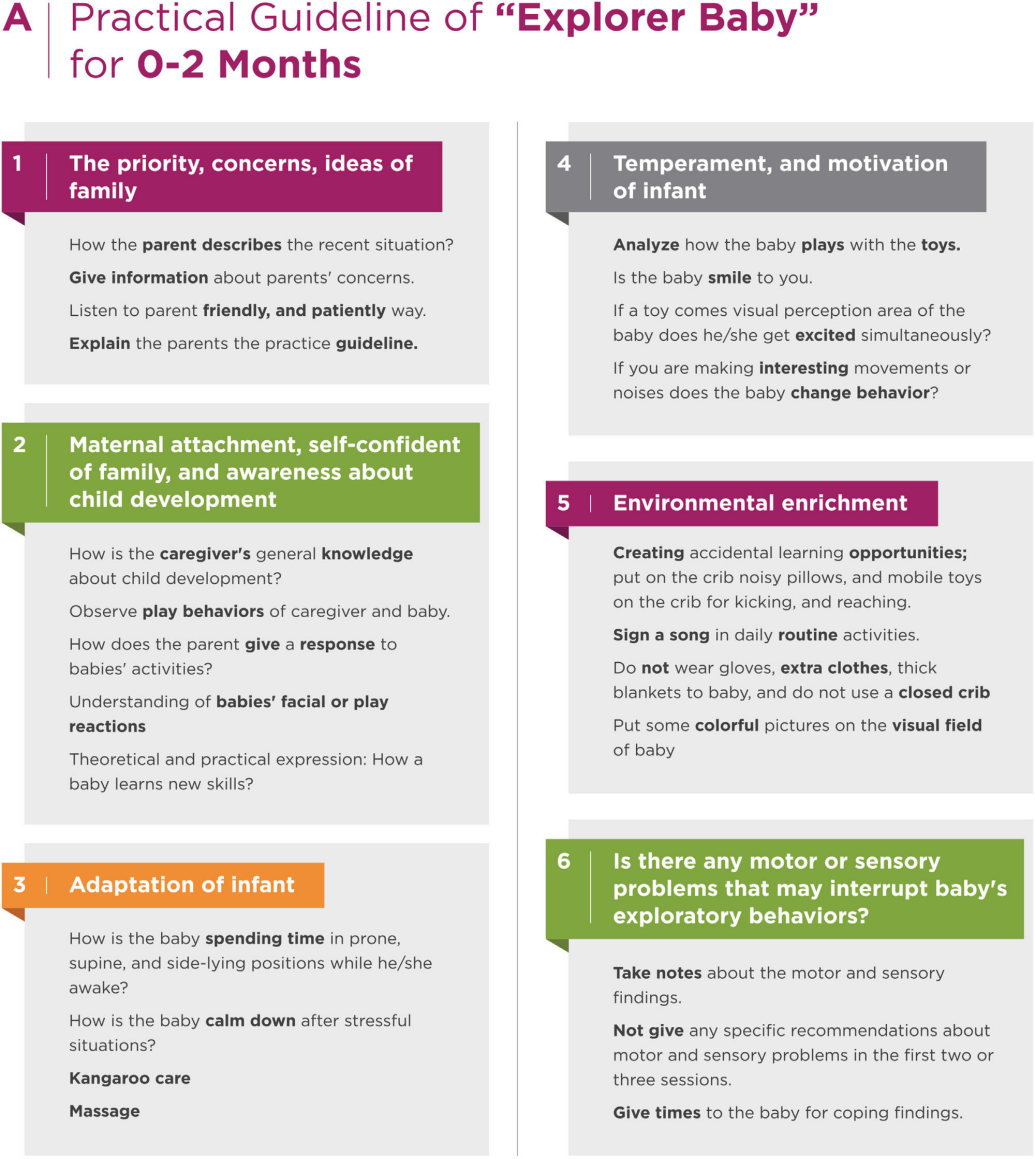

**Figure:**
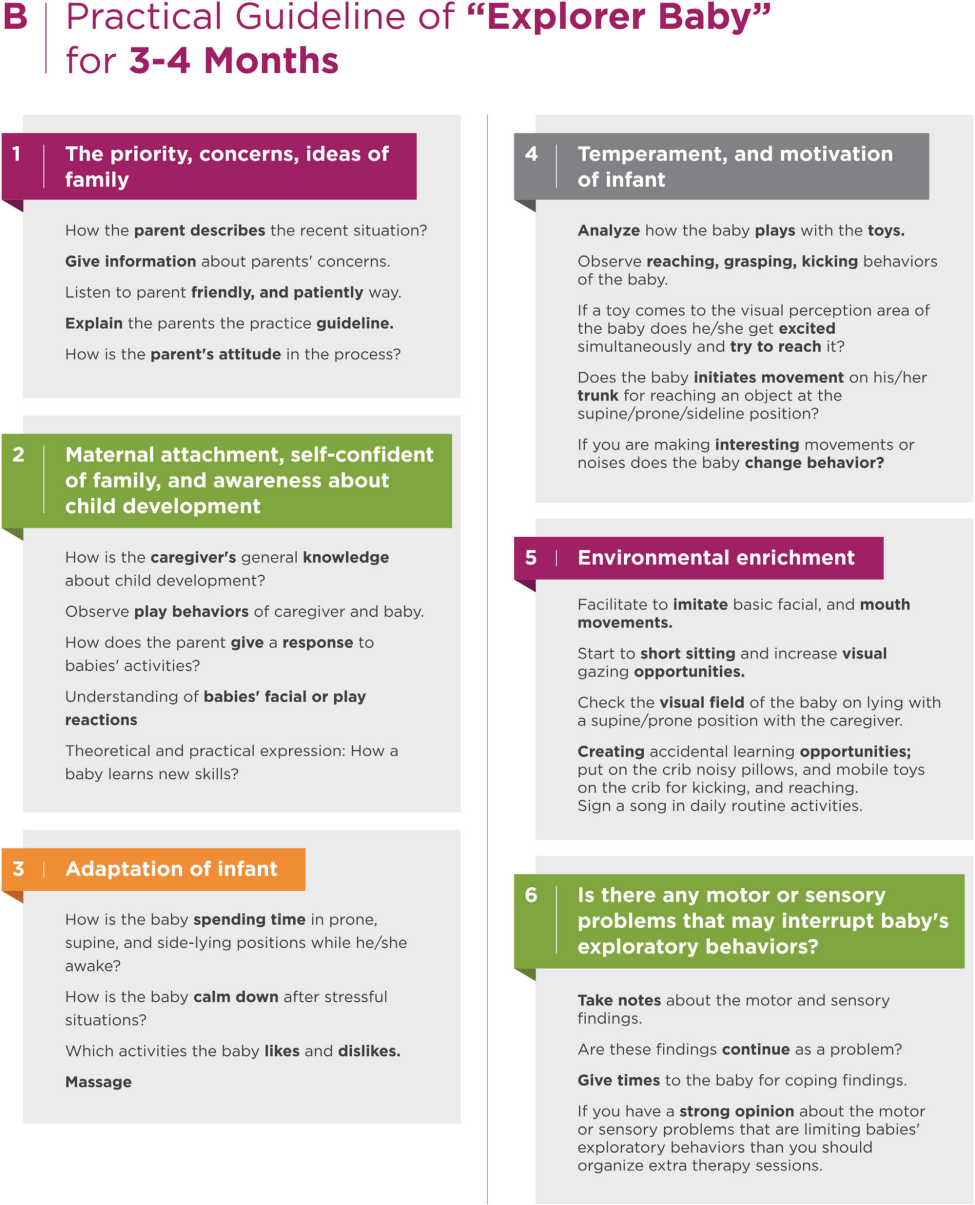

**Figure:**
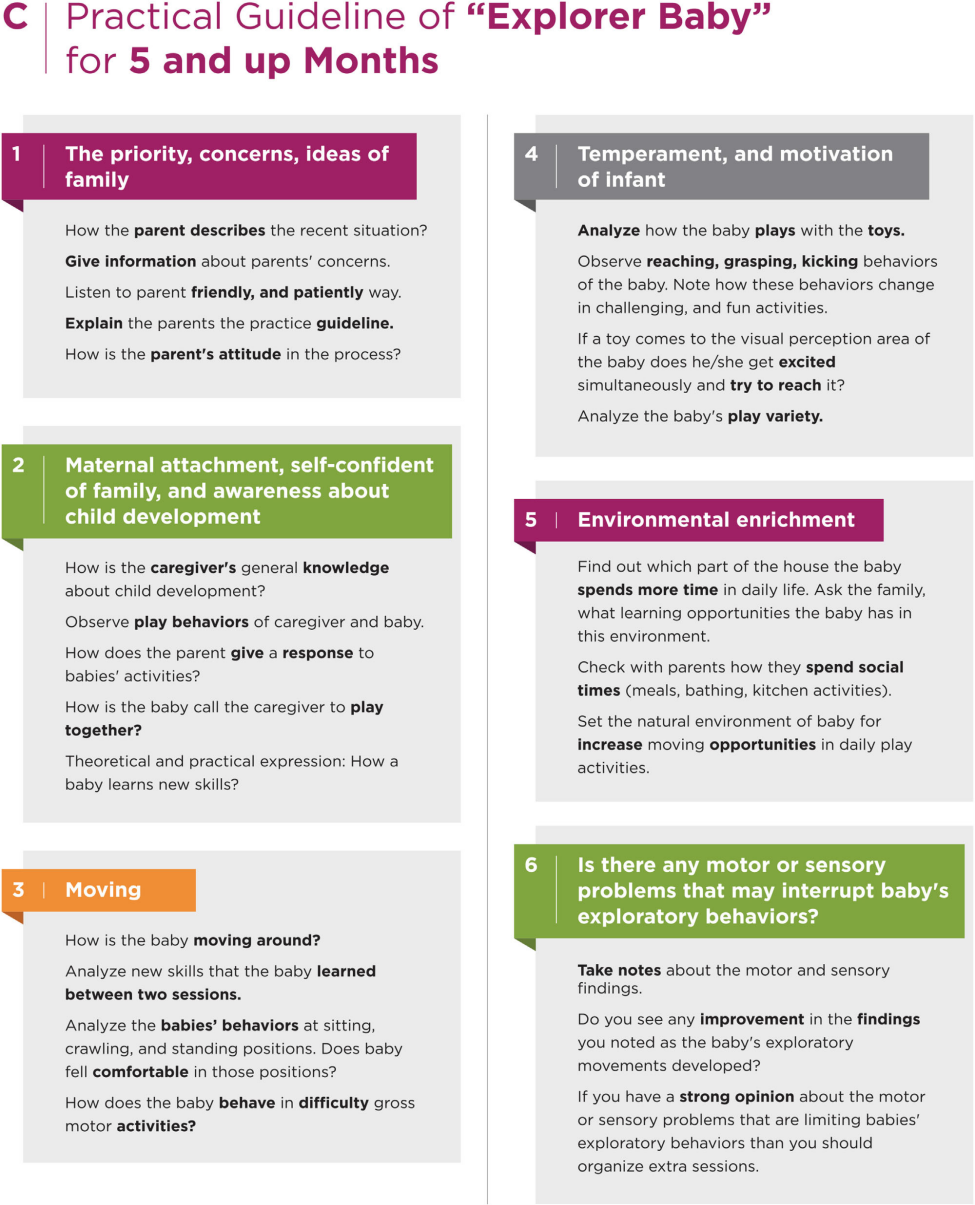

The Explorer Baby program contains kangaroo care, massage, and adaptation of different play positions, such as prone, supine, and side‐lying, improved parent‐baby interaction, and explorative play behaviors for the first 3 months of life. In the second 3 months of life, the Explorer Baby program highlights active playtime, explorative behaviors such as moving around, using both hands activities, kicking, reaching around, and self‐regulation (calming down or taking a rest during hard efforts).

#### 2.4.2 Neurodevelopmental therapy (NDT)

NDT is a concept aimed at identifying typical and atypical movements, facilitating typical movements, reducing atypical movements, and enhancing independence and function in daily activities. NDT practitioners undergo 40 days of training to learn facilitation techniques. These therapist‐guided techniques are called “handlings” or “hands‐on.” Handlings are special techniques used to prevent asymmetrical movements or atypical muscle tones in infants and children. The techniques were instructed for parents to use when holding, carrying, dressing, and bathing their children. The concept also uses positioning to encourage symmetrical movement and active participation of infants and children in daily activities such as play and feeding. NDT aims to improve function and participation by normalizing movement impairments. The concept aims to restore muscle tone and normal movement by suppressing atypical reflexes and postures (Vaughan‐Graham & Cott, 2016).

In our study, during the first 3 months of intervention, NDT had specific handling techniques for daily use, including carrying, bathing, changing diapers, and positioning the infant in the midline to maintain symmetrical movements throughout the day. It also uses U‐pillows and engaging toys in the visual space to help improve babies’ body control and facilitate their movement against gravity. In the second 3 months of intervention, the NDT included facilitation techniques for improving trunk control, moving against gravity, transferring weight among different positions, improving symmetrical movements, and improving play opportunities if it did not increase asymmetric or atypical movements. During both intervention periods, infants and children were kept midline and symmetrical with the help of cushions or blankets. The therapist encouraged child‐initiated movements if they fit typical movements (Girolami & Campbell, 1994). In this study, all these techniques were taught to parents during therapy by practicing, and they were asked to practice them regularly at home.

#### 2.4.3 Differences between Explorer Baby and NDT

The main difference between the two interventions is how physical therapists and parents work together. Although the physical therapist and parents share responsibilities in establishing the therapy program in the Explorer Baby program, the physical therapists decide on the therapy program and give a home program to parents in the NDT concept. The quality of movements is essential for the NDT, but trial and error processes are essential for the Explorer Baby. That means it is not important for the Explorer Baby program whether a child‐initiated movement occurs typically or atypically. Both programs aim to improve self‐initiated movements and play continuity, but NDT prioritizes improving more symmetrical and normal movements (File S2).

#### 2.4.4 Differences between Explorer Baby and current literature

In recent years, there have been several randomized controlled studies and systematic reviews in the field of early intervention, such as the Victorian Infant Brain Study (VIBeS Plus) (Spittle et al., 2009), Coping with and Caring for Infants with Special Needs (Akhbari Ziegler et al., 2019), and Supporting Play Exploration and Early Development Intervention (Dusing et al., 2018). The common points in these interventions are that the active participation of the parents in the intervention process and the child‐initiated movements are essential. The Explorer Baby program also recognizes the importance of these contents. The original aspect of the Explorer Baby program is that it aims to develop motor and social experiences through infants’ exploratory behaviors. The Explorer Baby program also has a cognitive component that supports infants in deciding whether or not to continue playing during contexts.

### 2.5 Outcomes

The participants were measured before (*T*0), during (*T*1 = *T*0 + 3 months), and after the therapy (*T*2 = *T*0 + 6 months). The natural development of infants in the first years is not linear. Some skills may develop fast, whereas others may develop slowly or stay stable. Therefore, we assessed participants at 3‐month intervals to state clearly the effect of therapies on the natural developmental curves of infants.

The other specific context of the first year of life is when an infant gains some skills in one subfield, such as gross motor development, which can adversely affect fine motor development (Valla et al., 2017).Thus, we reported all subfields with Bayley III to show the holistic effect of interventions. The study's primary results included the development of infant motor, cognitive, and language abilities (*T*0, *T*1, and *T*2), as well as an evaluation of parental stress levels (*T*0, *T*1, and *T*2) as a secondary outcome.

#### 2.5.1 *Bayley scales of infant and toddler development*, third edition (Bayley III)

Bayley III is a valid and reliable comprehensive assessment tool for children between the corrected ages of 15 days and 42 months. Bayley III evaluates cognitive, receptive language, expressive language, fine motor, and gross motor development. As Bayley III shows children's holistic development, we preferred to use it as a primary outcome measure. Bayley III includes raw scores, scale, composite, percentile, and growth scores. High points indicate better development. There are no developmental norms of Bayley III for the population of Türkiye. Therefore, we used the American term—children development norms. We reported Bayley III by using the scale score ranging from 1 to 19 points to give all subfields. We also used the composite score to identify children with and without developmental delays. We accepted the 85 composite scores to identify developmental delays, as it is recommended to use the cut‐off point for preterm infants (Albers et al., 2006).

#### 2.5.2 Parenting stress index‐short form IV (PSI‐SF‐4)

Parenting stress index‐short form IV measures the stress level of parents with children with special needs. The Türkiye validity and reliability study were conducted on parents who have children with special needs. In cases of multiple pregnancies, the mother was asked to rate only the baby she felt most stressed about. The questionnaire includes three domains: parental distress (PD), parent–child dysfunctional interaction (P‐CDI), and difficult child (DC). PD subheading includes stress related to a parent's perception of their child‐growing abilities, spousal dispute or level of assistance, and feelings of restraint related to their parental role. P‐CDI refers to stress occurring in parental feelings related to interaction with their child. DC means the parent's perspective on the difficulty of the child's temperament and demands. There are 36 questions to score for each item with a 5‐point Likert scale. The total points can vary from 36 to 180, and a low point indicates fewer stress behaviors. Thirty points and above are accepted as a high‐stress level for each subdomain (Cekic & Hamamci, 2018).

### 2.6 Sample size

The sample size was calculated with an 80% confidence interval using a 10% for minimally clinically significant Bayley III relevance using the G*Power program version 3.1.9.7. Supposing a 10% drop‐out rate, 25% infants in each group were required to ensure a statistical power of 80% chance of detecting this difference at a significance level of .05 (*α*). Therefore, we estimated the sample size needed to be 50 infants, 25 per group. According to repeated measures ANOVA on the total Bayley III scores, the partial eta squared is 0.204.

### 2.7 Statistical analysis

All the analyses were conducted using SPSS v24 (IBM Corp.). Participant characteristics were summarized for each treatment group with descriptive statistics. Two‐tailed tests (*a* = .05) evaluated any baseline differences between the groups. The results conformed to the normal distribution determined by the Shapiro–Wilk test. The current study had a repeated measurement design, so a linear mixed model with an unstructured covariance matrix was used with a random effect for the participants, fixed effects of time and the group, and interactions between time and the group to investigate the therapeutic effects. The effect size was calculated by Hedges’ *g* test when there was significance in the intragroup pre‐ and posttest comparisons. We performed repeated measures ANOVA and included all participants even if they had some missing data by using the “include the missing value” command via SPSS as the proportion of the missing data was less than 5% of all. Developmental delays were calculated over frequency and percentage and compared with Cochrane *Q* intragroup analysis. Cochran's *Q* test is a useful statistical tool for analyzing the differences in proportions between multiple related categorical variables, especially when the samples are dependent (Rosner, 2015).

## 3 RESULTS

### 3.1 Participant characteristics

Sixty‐eight participants were assessed for eligibility, and 57 took part in the trial. The participants were randomly divided into “Explorer Baby” (28 participants) and NDT (29 participants) groups. One infant from the NDT group withdrew from the study. At *T*2, two infants in the Explorer Baby group and three infants in the NDT group could not be evaluated because of the pandemic. In conclusion, we completed the study with 26 infants and 21 mothers in the Explorer Baby group, and 25 infants and 18 mothers in the NDT group (Figure 1). Demographic variables were homogeneously distributed between the two groups, except for bronchopulmonary dysplasia (BPD, *p* = .047). At baseline, the average age of the infants in the Explorer Baby group was 75.54 (days) corrected age, whereas the average age of the infants in the NDT group was 54.52 (days) corrected age. The baseline demographic and clinical characteristics are shown in Table 1.

**TABLE 1:** Baseline demographic and clinical characteristics of infants and parents.

Characteristic	Group 1 (28)	Group 2 (29)	*p*
Sex*, n* (%)			
Boy	16 (57.1)	11 (37.9)	.146^b^
Girl	12 (42.9)	18 (62.1)	
Age, mean (sd)	75.54 (42.59)	54.52 (40.66)	.227^a^
Birth week, mean (sd)	29 (2)	29 (2)	.389^a^
Birth weight, mean (sd)	1204 (314)	1321 (400)	.876^a^
Stay nicu, mean (sd)	61 (24)	50 (32)	.231^a^
Mother's age, mean (sd)	32.13 (4.96)	32.95 (5.42)	.179^a^
Father's age, mean (sd)	37.39 (5.07)	35.55 (6.49)	.285^a^
Mother's education, mean (sd)	10 (4)	11 (4)	.935^a^
Father's education	11 (4)	11 (4)	.643^a^
Cr findings, *n*			
No	22	27	.114^a^
Yes	6	2	
BPD*, n*			
No	12	18	.047* ^b^
Yes	7	2	
RDS*, n*			
No	5	4	.825^b^
Yes	18	17	
Income*, n*			
Minimum wage	15	16	.462^b^
Two times minimum wages	7	10	
Three and up times minimum wages	6	3	

*Note*: Group 1: Explorer Baby, Group 2: NDT.

Abbreviations: BPD, bronchopulmonary dysplasia; RDS: respiratory distress syndrome.

^a^ *p* Values were calculated using a Mann–Whitney *U* Test.

^b^ *p* Values were calculated using a Chi‐square independence test, *p≤.05.

### 3.2 The outcomes of infants’ development

#### 3.2.1 Between‐group analyses at *T*0, *T*1, and *T*2

**FIGURE 3:**
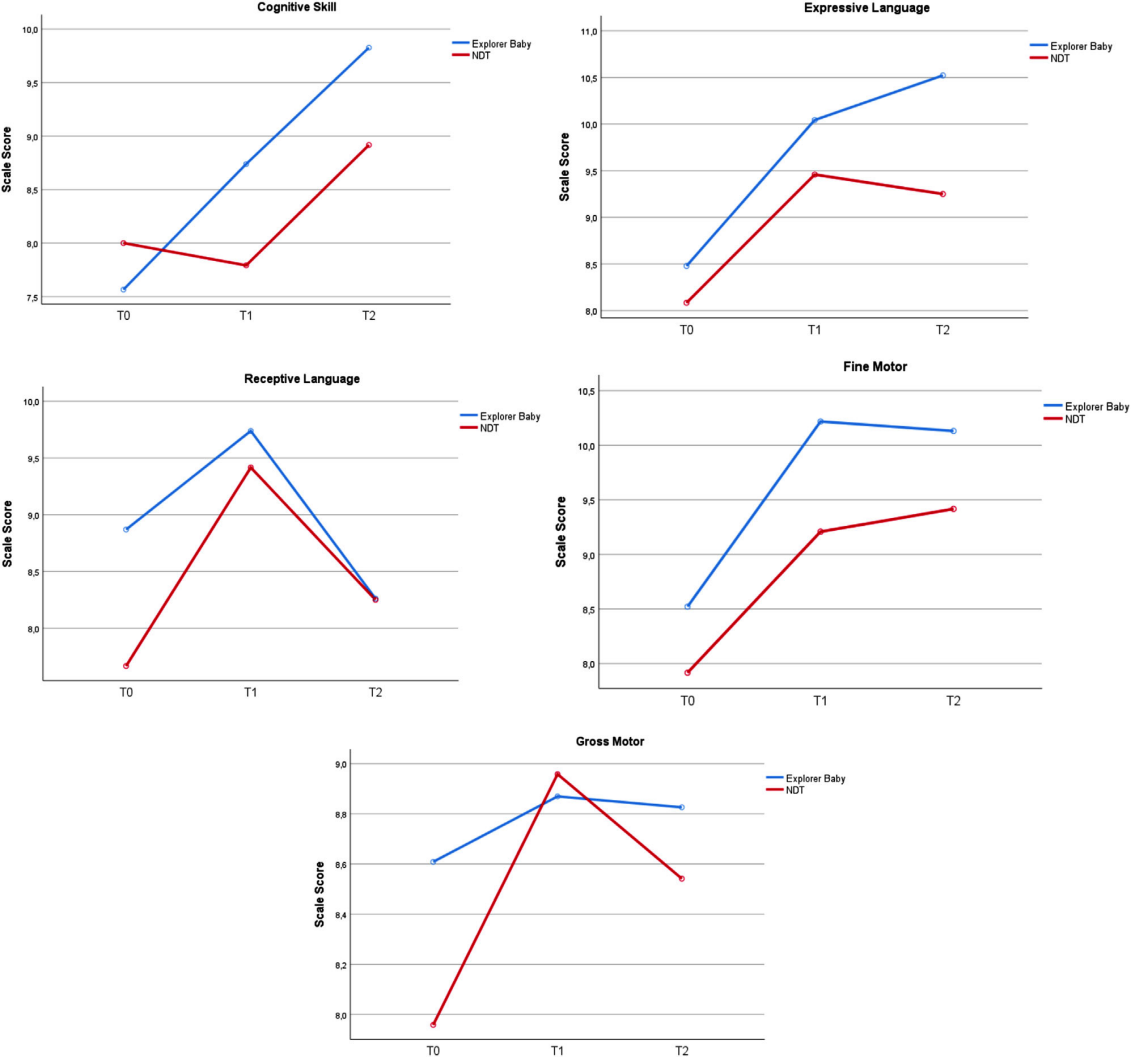
Developmental trajectories of two different intervention methods.

In the 6‐month developmental trajectories of preterm infants, no significant differences were observed in any parameters between the two intervention groups with (*p* > .05) and without (*p* > .05) a time effect (Table 2). Table 3 compares the percentages of developmental delays in cognitive, language, and motor skills between the two groups, and no significant difference was found between the two groups. Figure 3 compares the cognitive, language, and motor development curves of the groups over time.

**TABLE 2:** The Bayley III scale score before, during, and after therapy.

Variable	Group	T0	T1	T2	P time	P group	P time × group
Cognitive	Explorer	7.6 (3.1)	8.7 (2.8)	9.8 (2.0)	0.020^*^	0.232	0.345
	NDT	8.0 (2.7)	7.5 (3.0)	8.9 (2.9)	0.307		
Rec. lang.	Explorer	8.9 (3.3)	9.7 (2.4)	8.3 (2.4)	0.143	0.270	0.470
	NDT	7.7 (2.8)	9.4 (3.2)	8.3 (2.1)	0.094		
Expr. lang.	Explorer	8.5 (2.5)	10.0 (3.3)	10.5 (3.6)	0.041^*^	0.250	0.669
	NDT	8.1 (2.8)	9.5 (2.5)	9.3 (3.1)	0.105		
Fine motor	Explorer	8.5 (1.9)	10.2 (3.3)	10.1 (2.4)	0.074	0.109	0.927
	NDT	7.9 (1.7)	9.2 (3.1)	9.4 (3.0)	0.073		
Gross motor	Explorer	8.6 (1.8)	8.9 (2.5)	8.8 (3.6)	0.865	0.626	0.713
	NDT	8.0 (2.6)	9.0 (3.2)	8.5 (3.2)	0.323		

*Note*: *P* time: In‐group analyses (comparison before and after therapy for each group) with repeated measures ANOVA, *P* group: between‐group analyses, *P* time × group: between‐group analyses with controlling time effect, *T*0: before therapy, *T*1: during therapy, *T*2: after therapy, all assessments were based on corrected ages.

Abbreviations: Expr. lang.: expressive language; Rec. lang.: receptive language; NDT, neurodevelopmental therapy; *p≤.05.

**TABLE 3:** The percentage of developmental delays before during and after therapy (Bayley III composite score).

	*T*0		*T*1		*T*2		*Q* ^a^	*p*
	Delay *n* (%)	Typical *n* (%)	Delay *n* (%)	Typical *n* (%)	Delay *n* (%)	Typical *n* (%)		
Cog (EB)	8 (28.6)	20 (71.4)	6 (24.0)	19 (76.0)	0 (0.0)	26 (100.0)	9.46	.009* ^a^
Cog (NDT)	11 (37.9)	18 (62.1)	6 (21.4)	22 (78.6)	4 (16.0)	21 (84.0)	1.40	.497
Lang (EB)	8 (28.6)	20 (71.4)	4 (16.0)	21 (84.0)	4 (15.4)	22 (84.6)	2.17	.338
Lang (NDT)	11 (37.9)	18 (62.1)	5 (17.9)	23 (82.1)	7 (28.0)	18 (72.0)	1.86	.395
Motor (EB)	6 (21.4)	22 (78.6)	4 (16.0)	21 (84.0)	4 (15.4)	22 (84.6)	2.00	.368
Motor (NDT)	11 (37.9)	18 (62.1)	7 (25.0)	21 (75.0)	6 (24.0)	19 (76.0)	4.13	.127

*Note*: *T*0: before therapy, *T*1: during therapy, *T*2: after therapy, all assessments were based on corrected ages.

Abbreviations: Cog, cognitive; EB, explorer baby; lang., language; NDT, neurodevelopmental therapy.

^a^ Cochran's Q, *p≤.05.

#### 3.2.2 Within‐group analyses at *T*1 and *T*2

From *T*0 to *T*1, there was no significant change in any of the subskills. Both the Explorer Baby and the NDT groups presented varying degrees of beneficial effects across all developmental subskills when compared to *T*0 (Table 2). Table 3 compares the percentages of developmental delays in cognitive, language, and motor skills between the two groups, and no significant difference was found between the two groups.

From *T*0 to *T*2, The Explorer Baby group achieved statistically significant improvements in the cognitive (*p* = .020, Hedges’*g* = .83) and expressive language (Hedges’*g* = .65) subfields. Both groups maintain their improvements on the average score of developmental curves, except for receptive communication for Explorer Baby (Table 2). All infants in the Explorer Baby group showed normal development in cognitive skills at *T*2. This difference in cognitive skills compared to the baseline is statistically significant (*p* = .009) (Table 3).

### 3.3 The outcomes of maternal stress

From *T*0 to *T*2, no significant difference was observed in any parameter between the two groups with (*p* > .05) and without (*p* > .05) time effect on change in parental stress (Table 4).

**TABLE 4:** Therapy effect on maternal well‐being parenting stress index‐short form IV (PSI‐SF‐IV).

Variable	Group	*T*0	*T*1	*T*2	Time	Group	Time × group
PD	Explorer	28.11 (1.44)	26.89 (2.12)	26.63 (2.14)	0.643	0.892	0.979
	NDT	28.19 (1.57)	27.19 (2.31)	27.25 (2.33)	0.858		
P‐CDI	Explorer	24.21 (1.33)	24.89 (1.47)	24.21 (1.45)	0.864	0.496	0.069
	NDT	25.88 (1.45)	21.81 (1.60)	22.13 (1.58)	0.022*		
DC	Explorer	24.89 (1.46)	25.00 (1.55)	25.26 (1.55)	0.964	0.838	0.179
	NDT	27.63 (1.59)	24.25 (1.69)	24.44 (1.68)	0.094		
Total PSI	Explorer	77.21 (3.58)	76.79 (4.26)	76.11 (4.53)	0.944	0.933	0.274
	NDT	81.69 (3.90)	73.25 (4.64)	73.81 (4.94)	0.103		

*Note*: *P* time: In‐group analyses comparing before and after therapy, *P* group: between‐group analyses, *P* time × group: between‐group analyses with controlling time effect, *T*0: before therapy, *T*1: during therapy, *T*2: after therapy, all assessments were based on corrected ages.

Abbreviations: DC, difficult child; Expr. lang., expressive language; P‐CDI, parent–child dysfunctional interaction; PD, parental distress; Rec. lang., receptive language; NDT, neurodevelopmental therapy; *p≤.05.

In group the analyses, the NDT group exhibited significant improvement (*p* = .022, Hedges’ *g*: 2.66) in P‐CDI from *T*0 to *T*1 and *T*0 to *T*2. Total scores of parent stress levels were improved in both groups from *T*0 to *T*2 but were not statistically significant (Table 4).

### 3.4 Dose of intervention

Interventions were applied at different frequencies for 6 months according to the recommendations of each method. Infants in the Explorer Baby group received a total of 5.71 sessions during the 6‐month study period, whereas infants in the NDT group received a total of 5.91 sessions. There was no significant difference in the comparison of the therapy frequency between the two groups.

## 4 DISCUSSION

This is the first study to investigate the effect of the Explorer Baby early intervention program. We examined the holistic development of premature infants, the percentage of developmental delays, and the stress level of their mothers by comparing two early intervention programs during the first year of life. There were no significant differences between the intervention and active control groups in terms of the neurodevelopment of the preterm infants and their parents’ stress levels. However, we found that the Explorer Baby program significantly improved cognitive skills with a large effect and expressive language skills with a medium effect during before and after therapy. The percentage of typical development increased at the end of therapy in both groups, and all children had typical development in the Explorer Baby group in the cognitive subfield. The NDT group showed a significant improvement in P‐CDI during *T*1 and *T*2 with a large effect.

### 4.1 Group homogeneity

Parental demographics and infant biological risk factors were comparable in the two groups, with the exception of BPD variable. However, the Explorer Baby group had more infants with BPD than the NDT group. Literature shows that BPD is a risk factor for adverse developmental outcomes (Short et al., 2007). We applied stratified randomization in the gestational age and mother education, but controlling all parameters is impossible. Thus, it can be said that the Explorer Baby group was at a higher risk level of adverse outcomes than the NDT group.

### 4.2 Therapy effects for infants

During the first 3 months of intervention (*T*1–*T*0) in our study, the developmental trajectories of all subskills increased in both groups, but not significantly (Orton et al., 2009; Spittle et al., 2012, 2015), along with (Vanderveen et al., 2009), conducted systematic reviews and meta‐analyses to assess the impact of early intervention programs. These meta‐analyses indicate that early intervention programs have a small to medium effect on cognitive skills and a small effect on motor skills in infancy ages. Although meta‐analyses agree that early intervention in infants has benefits, some randomized controlled trials may show controversial results. Dusing et al. (2018) found a small beneficial effect in the intervention group on mean scores with no significant difference similar to our study. The control group in their study received active early intervention, as in our study, and not only vaccination or health‐child visits like main RCTs in meta‐analyses. This may be why there was no difference between the groups in Dusing et al. (2018) and our study. Fan et al. (2021) and Sgandurra et al. (2017) assessed the motor skills of preterm infants in their randomized controlled trials using two norm‐referenced tests: Gesell Developmental Schedules (GDS), Alberta Infant Motor Scales (AIMS) and two motor performance tests: Test of Infant Motor Performance (TIMP) and Infant Motor Profile (IMP). Results from these studies indicate that the TIMP and IMP assessments demonstrated significant motor changes, whereas motor skills on the GDS and AIMS did not change significantly. We used a norm‐referenced scale, which may have limited the detection of significant differences.

During the second period (corrected ages of 5–8 months) of our study (*T*2–*T*1), both groups showed a decline in trajectories for gross motor and receptive communication subfields, whereas other subskills either improved or remained stable. Sgandurra et al. (2017) indicated that skill acquisition has improved with increased time spent on treatment. Providing once a month therapy intensity for 3 months may not be frequent enough to achieve significant improvement in skills. Infants show more explorative behavior after the age of 5 months (Lobo et al., 2015) and acquire a range of motor skills, including independent sitting. Independent sitting is the primary function in the gross motor subfield between 5 and 8 months of age (Griffiths et al., 2018). Delayed independent sitting in premature infants (Gasparini et al., 2017) could explain the decrease in gross motor skills of both groups in our study. Apaydın et al. (2023) also examined the effectiveness of the SAFE technique in Turkish children and reported no significant changes in their gross motor and receptive language abilities. Additionally, Valla et al. (2017) demonstrated that gross motor and communication skills are the most heterogeneous skills in infants’ developmental trajectories during the first year of life, showing a “U” shape between 6 and 12 months. Therefore, it is likely that the mean scores of gross motor skills and receptive language skills decreased between *T*1 and *T*2 in both groups in our study. Surprisingly, Explorer Baby and NDT follow a similar trajectory in all developmental domains between *T*1 and *T*2, despite adopting different implementation principles. Interventions during early childhood can enhance the learning experience for children, even in the absence of strategies aimed at improving environmental enrichment or improving parent–child interaction (Vanderveen et al., 2009). Therefore, the development of the infants in the two groups may have progressed similarly from *T*1 to *T*2. Our study population included neurodevelopmentally low‐risk premature infants. This group has higher mobility than those with brain damage, which may have influenced the lack of difference between the two groups.

At the end of therapy (*T*2–*T*0), in our study, although both groups demonstrated varying degrees of improvement in all skills, there was no significant difference between the two groups. The cognitive outcomes of the Explorer Baby program align with the existing literature (Orton et al., 2009; Sgandurra et al., 2017). This can be explained by the fact that Explorer Baby, like current early intervention programs, is an approach to developing new skills through environmental enrichment, active experiences, and parent–child interaction. Although major premature infants follow a normal developmental curve, the risk of developmental delay in cognitive, motor, and language areas can increase by up to 10 times. Preterm infants may experience various negative consequences, such as cognitive, motor, language, and social impairments (Aylward, 2014; Kara et al., 2020; Ment et al., 2003), and their natural developmental trajectory may lag behind that of full‐term infants (Ferreira R de et al., 2020; Gasparini et al., 2017). However, in our study, the average scores for all skills increased in both groups and the number of children with developmental delays decreased. We interpreted these improvements in both groups as clinically significant, although not statistically. No significant difference was detected in any skill between Explorer Baby and NDT. As significant improvements were found in cognitive and expressive language in within‐group analyses, it can be said that the beneficial effect was higher in Explorer Baby. The frequency of therapy in early intervention is a critical issue for applicability and dissemination. As the premature birth rate is as high as 12%, even developed countries may find it difficult to pay for early intervention programs more frequently than once a month (Beck et al., 2010). Furthermore, parents of premature infants also prefer a frequency of 1 per month in early intervention programs (Mobbs et al., 2022). We believe that the Explorer Baby program is a cost‐effective approach with a monthly application frequency, as it has a beneficial effect in multiple subfields (but not significantly) on all areas of development. For national systems that support premature infants, interventions promoting comprehensive development, such as Explorer Baby with one session per month, may be more clinically relevant than interventions that solely promote motor or cognitive skills once a week.

### 4.3 Therapy effects for parents

There was no significant difference between the two groups in any field of maternal stress at *T*0, *T*1, and *T*2. All sub‐stress scores and total scores improved at the end of therapy in both groups, except for the DC subfield in the Explorer Baby group. Girabent‐Farrés et al. (2021) have provided moderate‐to‐strong evidence that early intervention reduced the stress of mothers with preterm infants regardless of the method. Parallel to the literature, our study found that both groups showed improvement in total parental stress scores, although this was not statistically significant. Parental stress positively correlates with neurodevelopmental outcomes (Erdei et al., 2021; Wu et al., 2014). Due to the significant improvement in cognitive and expressive language subskills of the Explorer Baby group between the pre‐ and posttest, it was expected that maternal stress scores of the Explorer Baby group would also improve. NDT group showed significant improvement in P‐CDI between *T*0–*T*1 and *T*0–*T*2. Explorer Baby program encourages parents to take responsibility for providing an optimal learning environment. Moreover, expert suggestions do not include specific directives because parents are motivated to behave depending on the infant's play behaviors and the natural context of that day. These open‐ended responsibilities may increase maternal stress in the DC subfield. Future research should investigate the maternal preferences for sharing responsibilities in early intervention practices. Another explanation for this finding is the fact that the increased mother's stress level in the DC may be because the Explorer Baby program improved the parents’ awareness to observe the children's behavior and emotional states.

### 4.4 Limitations

The current study had some limitations. Explorer Baby is a family‐centered practice, but we do not have assessment tools to measure family‐centeredness. Future studies should examine the effectiveness of the Explorer Baby program in natural environments. We investigated only maternal stress, and we suggest that future studies also investigate paternal stress. Another limitation was that we did not have a third group receiving standard care, so it was more difficult to interpret the therapeutic effect of interventions. We purposely targeted children at high risk for developmental delays and low risk for Cp because we aimed to show the holistic effect of the intervention not only gross motor subskill. We compared the efficacy of both methods at a frequency of 1 therapy per month. This frequency of therapy may not have been sufficient to significantly differentiate the treatment effect. Further research should investigate the efficacy of the Explorer Baby program at different levels of intensity.

## 5 CONCLUSION

Preterm infants are at a higher risk of developmental delays when compared to their term‐born peers. Early intervention programs are applied to reduce these adverse outcomes. Explorer Baby and NDT programs enhance the development of preterm infants and improve the total stress levels of mothers. There is no significant difference between the two groups regarding infants’ development and parental stress. Both methods improved the developmental trajectories of premature infants and reduced overall parental stress, but not to a statistically significant degree. Both methods can be implemented as early intervention programs because they showed beneficial effects on the developmental trajectories of preterm infants and overall parental stress. The Explorer Baby program may be a preferred option for supporting neurodevelopment in preterm infants, as the program showed greater skill acquisition (not significant) and did not place an additional burden on parents’ overall stress levels. The Explorer Baby program is also well suited for national early intervention systems that prefer a frequency of 1 per month. These results encourage further studies to examine the effectiveness of the Explorer Baby early intervention program in different diagnostic groups, at different intensities, home visits, telerehabilitation, and well‐child visits. We recommend the use of both norm‐referenced and motor performance assessments in early intervention research related to the first year of life.

## PATIENT CONSENT STATEMENT

Primary caregivers were informed, and their verbal and written consents were obtained before the initial assessment.

## CLINICAL TRIAL REGISTRATION

The National Clinical Trial number is NCT04203589.

## CONFLICTS OF INTEREST STATEMENT

The authors declare no conflicts of interest.

### PEER REVIEW

The peer review history for this article is available at https://publons.com/publon/10.1002/brb3.3266.

## Supporting information

Supplementary File 1

Supplementary File 2

## Data Availability

Study data will be made available upon reasonable request to the corresponding author. De‐identified data will be made available upon reasonable request.
