# Supplementary material for: Early developmental support for preterm infants based on exploratory behaviors: A parallel randomized controlled study
Source: Brain Behav. 2023 Oct 5;13(11):e3266. doi: 10.1002/brb3.3266 (PMC10636377; doi:10.1002/brb3.3266)
Supplement: Supplementary file 1 [file BRB3-13-e3266-s002.docx]

**
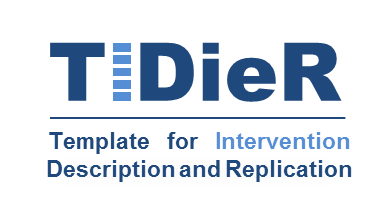
The TIDieR (Template for Intervention Description and Replication) Checklist*:**

Information to include when describing an intervention and the location of the information

| **Item number** | **Item** | **Where located **** | |
| --- | --- | --- | --- |
|  |  | Primary paper  (page or appendix  number) | Other ^†^ (details) |
|  | **BRIEF NAME** |  |  |
| **1.** | “Explorer Baby” (**EXPLOR**atory behaviors based early intervention) | P1 |  |
|  | **WHY** |  |  |
| **2.** | From the moment they are born, babies are ready to interact with the environment, learn and develop. We already know that learning is a dynamic process gained through variable experiences between individuals and the environment (Smith & Thelen, 1994). The high number of exploratory motor movements indicates that the baby has much experience. The Explorer Baby program aims to increase exploratory motor behaviors by considering the individual differences of babies. | P2 |  |
|  | **WHAT** |  |  |
| **3.** | The intervention group received written documentation. During the sessions, each family was given a written document describing the intervention according to the baby's corrected age. For example, the family of the corrected 5-month-old baby was provided a previously prepared 5-month-old baby document.  Age-specific activities and environmental arrangements were made with parents during the sessions. All materials used in the sessions were tools that families could find at home (refrigerator bag, gift wrap, rattle, pillow, box, etc.). | P3 |  |
| **4.** | The “Explorer Baby” early intervention program includes six major themes for the first six months of life:   1. The priorities, concerns, and ideas of parents/caregivers 2. Maternal-infant attachment, the parents’ awareness and self-confidence of child development 3. Environmental adaptation of an infant 4. Temperament and motivation of an infant 5. Environmental enrichment 6. Managing motor or sensory problems that may interrupt the baby's exploratory behaviors.   Each section has specific aims, activities, and written documentation.  Critical elements for “Explorer Baby.”  **Family-centered practice:** The first two of these six items in the program are related to family-centered approaches. All therapy sessions start with an explanation of the current situation by the parents. The parents are motivated to express their priorities, concerns, and feelings. The professional and parents observe the baby’s play behaviors together. Family-centered practices provide an implementation guide for collaborating with parents. Working with parents concerning what they feel and aiming to improve their participation in their daily routine is accepted as a part of the family-centered practice. The family's natural environment consists of everyday experiences that may affect all the family members' physical and mental health. In this perspective, the Explorer Baby program aims to increase exploratory behaviors and positive temperament of infants with bidirectional communication with parents.  The professional asks parents;   - What your baby may be learning during these play experiences. - How do you think your baby is feeling right now? - Do you think your baby wants to continue playing? - What can we do to improve your baby's playtime?   After parents respond, the therapist starts to explain why these questions are essential for learning new skills. This step aims to improve the knowledge and skills of parents about child development.   - The therapist observes the play behaviors and communication between the infant and the parent. Then ask new questions to parents; - How do you feel playing with your baby? - How do you notice your baby's play preferences? - Did you notice the experiences during the game (this section refers to actual experiences during the game)? - You know how these experiences will benefit your baby and you, right?   **Adaptation, temperament, and motivation of an infant:**  The temperament of infants is an essential factor that affects how they behave in the play environment. The character can be described as play motivation, adaptation, self-regulation, activity skills, and exploratory behaviors. Infants with positive temperament characteristics should be expected to participate in environmental play opportunities. They should be expected to develop more rapidly than infants who do not have the chance to explore. The Explorer Baby program supports the positive temperament of infants by creating opportunities to exhibit their exploratory behaviors in activities where infants can be successful with little difficulty (The Zone of Proximal Development).  “Explorer Baby” early intervention program is based on exploratory behaviors. Infants' environmental adaptation and personal characteristics have a specific role in exploratory behaviors. The aims of the program for exploratory behaviors are below;   - To improve the environmental adaptation of infants in different play positions (supine, prone, side-lying, and sitting). - To improve the motivation of play, social interaction, and engagement in environmental events. - To improve play variability and playtime.   **Environmental enrichment:**  The program has been developed based on dynamic system theories and neuronal group selection theories. The program uses environmental enrichment to increase play opportunities. In addition, the program tries to increase the trial-and-error behaviors in these plays by developing exploratory behaviors.  **Managing motor or sensory problems that may interrupt baby's exploratory behaviors:**  Body structural difficulties (such as hypotonia and hypertonia) can affect infants' play preferences. However, the Explorer Baby program believes babies will challenge many body structure problems during their daily play experience. Therefore, even if an Explorer Baby practitioner detects body structure problems, it allows babies to solve these problems for a while (one or two months).  **Learning with trial-and-error process:**  Parents of premature babies are much more responsive to their baby's care. This can sometimes negatively affect the baby's an exploration and play process. For example, 4-month-old babies often try to roll over. They usually do this while reaching for the toy they want to pick up. This trial-and-error process is skipped when the distant toy is immediately given to the baby. The program informs the family about factors that may limit the trial-and-error process of the baby.  **Therapy sessions:**  The appointments of the sessions are determined according to the baby's routine and the parent's preference. | P3 and P5 |  |
|  | **WHO PROVIDED** |  |  |
| **5.** | Two physiotherapists who developed the “Explorer Baby” early intervention program served individual sessions. One physiotherapist had more than 15 years of experience in pediatric rehabilitation, while the other had ten years of experience. The therapists implement the program together for a week to follow the same protocol. | P 5 |  |
|  | **HOW** |  |  |
| **6.** | All intervention was provided face-to-face on an individual basis. Intervention practitioners followed the standard protocol of the “Explorer Baby” early intervention program. The program has age-specific implementation documents. The therapist followed the age-specific document—at the end of the therapy, this document was given to the parent. | P 5 |  |
|  | **WHERE** |  |  |
| **7.** | “Explorer Baby” was provided at the family consultation center. This center is affiliated with a nonprofit NGO. It is located in a metropolitan area and has different public transportation options. | P 5 |  |
|  | **WHEN and HOW MUCH** |  |  |
| **8.** | “Explorer Baby” was offered at least monthly; however, some infants had more frequent sessions during the study period. Sessions were typically forty-five minutes in length. Physiotherapists offered therapy more than once per month according to the needs. For examples;  If a parent has stressful behaviors  If the parents need more self-confidence about general knowledge of child development.  If the parents say they need more meetings/discussions with the professional  If the professional thinks that the parents need more sessions to get the idea  If the baby has Body structural difficulties | P 5 |  |
|  | **TAILORING** |  |  |
| **9.** | Every infant has a physiotherapist. The same therapist followed most babies, but different therapists followed some. | P 5 |  |
|  | **MODIFICATIONS** |  |  |
| **10.^ǂ^** | Several infants were noted to have Cerebral Palsy signs. The infant dropped from the study and enrolled in another project when this occurred. In addition, several infants were reported to have hypotonia and sensory issues, which limitate to their exploratory behaviors. In this situation, the sessions are organized more frequently. | P 5 |  |
|  | **HOW WELL** |  |  |
| **11.** | The two therapists followed three infants together before the program started. In addition, the therapists occasionally attended each other's sessions as observers. | P 5 |  |
| **12.^ǂ^** | All sessions were recorded on the camera. Therapists continued to watch each other's session records throughout the study. | P 5 |  |

** **Authors** - use N/A if an item is not applicable for the intervention being described. **Reviewers** – use ‘?’ if information about the element is not reported/not sufficiently reported.

† If the information is not provided in the primary paper, give details of where this information is available. This may include locations such as a published protocol or other published papers (provide citation details) or a website (provide the URL).

ǂ If completing the TIDieR checklist for a protocol, these items are not relevant to the protocol and cannot be described until the study is complete.

* We strongly recommend using this checklist in conjunction with the TIDieR guide (see *BMJ* 2014;348:g1687) which contains an explanation and elaboration for each item.

* The focus of TIDieR is on reporting details of the intervention elements (and where relevant, comparison elements) of a study. Other elements and methodological features of studies are covered by other reporting statements and checklists and have not been duplicated as part of the TIDieR checklist. When a **randomised trial** is being reported, the TIDieR checklist should be used in conjunction with the CONSORT statement (see [www.consort-statement.org](http://www.consort-statement.org)) as an extension of **Item 5 of the CONSORT 2010 Statement.** When a **clinical trial** **protocol** is being reported, the TIDieR checklist should be used in conjunction with the SPIRIT statement as an extension of **Item 11 of the SPIRIT 2013 Statement** (see [www.spirit-statement.org](http://www.spirit-statement.org)). For alternate study designs, TIDieR can be used in conjunction with the appropriate checklist for that study design (see [www.equator-network.org](http://www.equator-network.org)).
