# Supplementary material for: Early developmental support for preterm infants based on exploratory behaviors: A parallel randomized controlled study
Source: Brain Behav. 2023 Oct 5;13(11):e3266. doi: 10.1002/brb3.3266 (PMC10636377; doi:10.1002/brb3.3266)
Supplement: Supplementary file 2 [file BRB3-13-e3266-s001.docx]

**
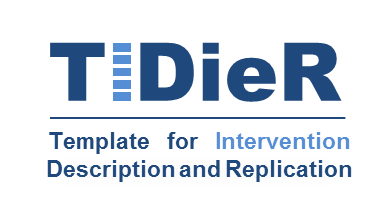
The TIDieR (Template for Intervention Description and Replication) Checklist*:**

Information to include when describing an intervention and the location of the information

| **Item number** | **Item** | **Where located **** | |
| --- | --- | --- | --- |
|  |  | Primary paper  (page or appendix  number) | Other ^†^ (details) |
|  | **BRIEF NAME** |  |  |
| **1.** | NeuroDevelopmental Therapy-Bobath (NDT-Bobath) | P 1 |  |
|  | **WHY** |  |  |
| **2.** | NDT underlies the importance of motor learning based on a child’s activity. During the child’s activity, NDT aims to increase movement and functional abilities in more typical ways. For example, handling techniques are used to improve the variety of movements and change atypical postures. As the baby experiences movements in a more quality way, it is thought that this will enhance their functional skills. | P 6 |  |
|  | **WHAT** |  |  |
| **3.** | The control group received a handling and positioning training program. The handling and positioning training program is unique for every baby according to their special needs. The physiotherapists showed the practices to the parents and asked them to do them. | P 6 |  |
| **4.** | Body structure problems such as Hypotonia in the trunk are common in premature babies. To reduce the effect of hypotonia on the baby, a U pillow was used when the baby was lying supine. It was provided to facilitate the skills such as coming to the midline, reaching out to the toys, doing chin-tuck, and keeping the baby more organized. Parents used it as a U pillow by folding their sheets or blankets at home. Similar equipment extracted qualified and typical movements and reduced atypical activities. Experts explained to parents why and how to use the equipment at home.  Handling techniques were used to facilitate typical movements, increase the movement variations of the baby, and interact with the environment and toys. Experts practiced the techniques, showed them to the parents, and then they did it together. | P 6 |  |
|  | **WHO PROVIDED** |  |  |
| **5.** | Two physiotherapists who both are certified NDT therapists (certified from European Bobath  Tutors' Association-EBTA) served individual sessions. One physiotherapist had more than 25 years of experience in pediatric rehabilitation, while the other had eight years of experience. The more experienced physiotherapist is also Bobath Tutor in Turkey with EBTA certification. At the beginning of the study, the therapists served sessions together to follow the same protocol. | P 5 |  |
|  | **HOW** |  |  |
| **6.** | All intervention was provided face-to-face on an individual basis. Intervention practitioners followed the standard protocol of the “Explorer Baby” early intervention program. The program has age-specific implementation documents. The therapist followed the age-specific document. At the end of the therapy, this document was given to the parent. | P 6 |  |
|  | **WHERE** |  |  |
| **7.** | “NDT” was provided at the family consultation center. This center is affiliated with a nonprofit NGO. It is located in a metropolitan area and has different public transportation options. | P 3 |  |
|  | **WHEN and HOW MUCH** |  |  |
| **8.** | “NDT was offered at least monthly, and some infants had more frequent sessions during the study period. Sessions were typically forty-five minutes in length. In addition, physiotherapists offered therapy more than once per month according to the needs. | P 5 |  |
|  | **TAILORING** |  |  |
| **9.** | Every infant has a physiotherapist. The same therapist followed most babies, but different therapists followed some. Each intervention program was planned according to the infant’s and the family’s needs. For example, if the infant has other special needs, such as visual impairment, these were added to the intervention plan. |  |  |
|  | **MODIFICATIONS** |  |  |
| **10.^ǂ^** | Several infants were noted to have Cerebral Palsy signs. The infant dropped from the study and enrolled in another project when this occurred. In addition, several infants were reported to have tone problems than more frequent sessions planned for them. |  |  |
|  | **HOW WELL** |  |  |
| **11.** | NDT is a standard method that can be applied with a certificate. Some practice sessions were recorded on video. |  |  |
| **12.^ǂ^** |  | P5 |  |

** **Authors** - use N/A if an item is not applicable for the intervention being described. **Reviewers** – use ‘?’ if information about the element is not reported/not sufficiently reported.

† If the information is not provided in the primary paper, give details of where this information is available. This may include locations such as a published protocol or other published papers (provide citation details) or a website (provide the URL).

ǂ If completing the TIDieR checklist for a protocol, these items are not relevant to the protocol and cannot be described until the study is complete.

* We strongly recommend using this checklist in conjunction with the TIDieR guide (see *BMJ* 2014;348:g1687) which contains an explanation and elaboration for each item.

* The focus of TIDieR is on reporting details of the intervention elements (and where relevant, comparison elements) of a study. Other elements and methodological features of studies are covered by other reporting statements and checklists and have not been duplicated as part of the TIDieR checklist. When a **randomised trial** is being reported, the TIDieR checklist should be used in conjunction with the CONSORT statement (see [www.consort-statement.org](http://www.consort-statement.org)) as an extension of **Item 5 of the CONSORT 2010 Statement.** When a **clinical trial** **protocol** is being reported, the TIDieR checklist should be used in conjunction with the SPIRIT statement as an extension of **Item 11 of the SPIRIT 2013 Statement** (see [www.spirit-statement.org](http://www.spirit-statement.org)). For alternate study designs, TIDieR can be used in conjunction with the appropriate checklist for that study design (see [www.equator-network.org](http://www.equator-network.org)).
